# Changes in menstrual symptoms and work productivity after checklist-based education for premenstrual syndrome: an 8-month follow-up of a single-arm study in Japan

**DOI:** 10.1186/s12905-024-03067-2

**Published:** 2024-04-15

**Authors:** Chihiro Ozeki, Eri Maeda, Osamu Hiraike, Kyoko Nomura, Yutaka Osuga

**Affiliations:** 1 Department of Environmental Health Science and Public Health, Akita University Graduate School of Medicine, Akita, Japan; 2 Department of Public Health, Graduate School of Medicine, Hokkaido University, Kita 15, Nishi 7, Kita-Ku, Sapporo, 060-8638 Japan; 3 Department of Obstetrics and Gynecology, Graduate School of Medicine, The University of Tokyo, Tokyo, Japan

**Keywords:** Premenstrual syndrome, Educational intervention, Medical help-seeking, Work productivity

## Abstract

**Background:**

Premenstrual syndrome (PMS) is prevalent among women of reproductive age, but most do not seek medical advice. We hypothesized that building PMS awareness could promote medical help-seeking for PMS and thus reduce menstrual symptoms and improve work productivity.

**Methods:**

In January 2020, women aged between 25 and 44 years, having paid work, and not currently consulting with an obstetrics and gynecology doctor (*n* = 3090) responded to the Menstrual Distress Questionnaire (MDQ), the Premenstrual Symptoms screening tool, and the World Health Organisation Health and Work Performance Questionnaire. In addition, they received checklist-based online education for PMS. Of 3090 participants, 2487 (80.5%) participated in a follow-up survey in September 2020. We conducted multiple logistic regression analyses and text analyses to explore factors that encouraged and discouraged medical help-seeking. We also evaluated changes in menstrual symptoms and work productivity, using generalized estimating equations with interactions between the severity of PMS, help-seeking, and time.

**Results:**

During the follow-up period, 4.9% of the participants (121/2487) sought medical help. Those having high annual income (adjusted odds ratio [aOR] = 2.07, 95% confidence interval [CI]: 1.21–3.53) and moderate-to-severe PMS (aOR = 2.27, 95% CI: 1.49–3.46) were more likely to have sought medical help. Those who did not seek medical help despite their moderate-to-severe PMS reported normalization of their symptoms (36%), time constraints (33%), and other reasons for not seeking medical help. Participants with moderate-to-severe PMS who had sought medical help showed a significant improvement of − 8.44 points (95% CI: − 14.73 to − 2.15 points) in intermenstrual MDQ scores during the follow-up period. However, there were no significant improvements in premenstrual and menstrual MDQ scores or absolute presenteeism.

**Conclusion:**

Medical help-seeking alleviated intermenstrual symptoms in women with moderate-to-severe PMS, but only a small proportion of them sought medical help after PMS education. Further research should be conducted to benefit the majority of women who are reluctant to seek medical help, including the provision of self-care information.

**Trial registration:**

UMIN Clinical Trials Registry number: UMIN000038917.

**Supplementary Information:**

The online version contains supplementary material available at 10.1186/s12905-024-03067-2.

## Background

Premenstrual syndrome (PMS) is characterised by a combination of cyclical physical or mood symptoms, or both, that occur discretely during the luteal phase and resolve during or shortly after the menstrual period [1]. According to the clinical practice guideline by the American College of Obstetricians and Gynecologists [1], the most common symptoms are irritability, bloating, mood swings, lethargy, breast tenderness, anxiety and tension, and feelings of rejection. Appendix shows some common PMS symptoms [2]. A recent systematic review of 17 studies worldwide found that the pooled prevalence of PMS was 48%, with an increasing trend over time and a wide range of prevalence between studies [3]. In Japan, the prevalence of moderate to severe PMS and premenstrual dysphoric disorder (PMDD) (i.e., the severe subtype of PMS) was 18% among female college students [4]. Many PMS patients may benefit from a comprehensive strategy that combines multiple interventions based on shared decision-making between patients and clinicians, including pharmacologic agents, psychological counseling, complementary and alternative treatments, exercise and nutritional therapies, patient education and self-help strategies, and surgical management [1, 5, 6].

Despite the benefits of PMS treatment, a previous study in Japan revealed that only 5% of women with moderate to severe PMS and PMDD received medical treatment [7]. Japanese women are generally considered to be reluctant to receive treatment for gynecological and reproductive health concerns; for example, the lifetime prevalences of oral contraceptive use and hormone replacement therapy use were as low as 6.0% and 13.8%, respectively, in Japanese nurses [8]. In addition, a recent survey conducted by a biotech company found that only 10% of women have a primary care obstetrics and gynecology doctor, even though more than half of those surveyed felt they needed one [9]. Thus, it is likely that many women do not receive appropriate treatment for PMS.

PMS can affect work productivity, as well as quality of daily life. Women with PMS reported reduced work productivity and more work days missed for health reasons [10]. High-school students with PMS were more likely to lack concentration and motivation and to have poorer academic performance [11]. Although the economic burden associated with PMS has not been estimated in Japan, work productivity loss resulting from menstrual symptoms reaches 491 billion JPY annually [12]. Given the growing number of women joining the labor force [13], it is crucial to promote awareness that seeking appropriate medical help could reduce the symptoms of PMS and result in greater work productivity.

**Fig. 1:**
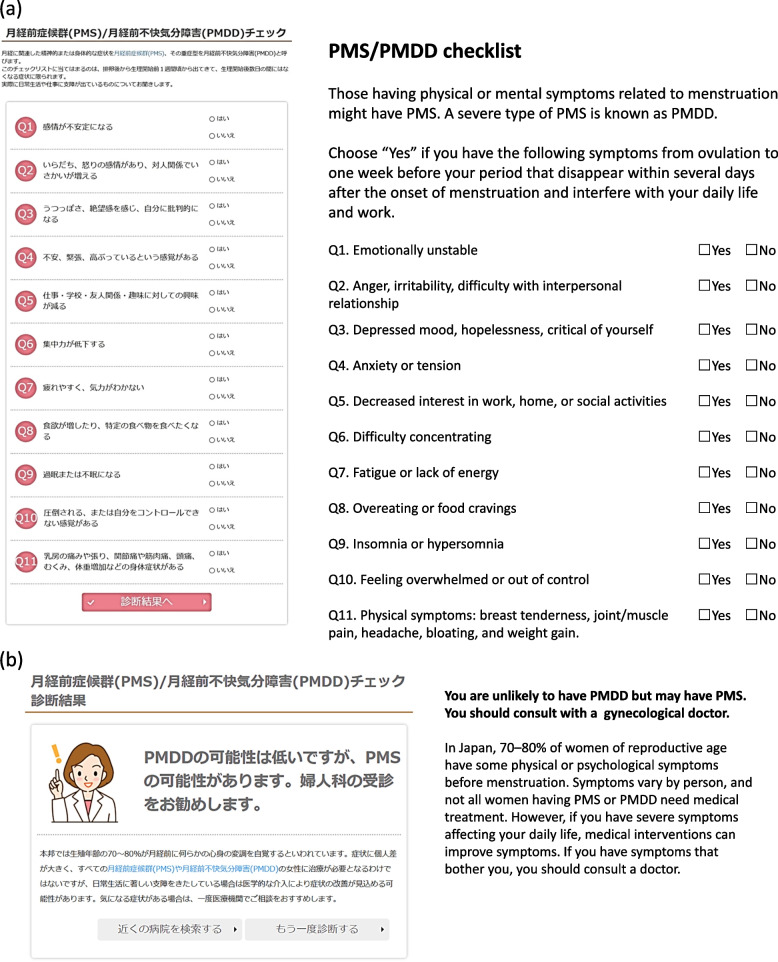
The checklist-based online educational tool for premenstrual syndrome. PMS, premenstrual syndrome; PMDD, premenstrual dysphoric disorder (**a**) PMS/PMDD checklist (**b**) An example of advice automatically shown for women who ticked two symptoms

To promote women’s health, obstetrics and gynecology professionals in Japan launched a government-supported website in 2016 (https://w-health.jp/). The website provides educational information to the public, covering biopsychosocial health topics from puberty to menopause. Users can learn about PMS by using a checklist form and clicking whether they have each typical symptom (Fig. 1a). The results could encourage those who might have PMS to consult with a doctor (Fig. 1b). However, it is unknown whether such awareness initiatives for PMS promote appropriate help-seeking behaviors or improve symptoms. Thus, this study aimed to assess the effectiveness of PMS awareness initiatives on women’s help-seeking behaviors, symptoms, and work productivity. We recruited women of reproductive age who had paid work and were not currently consulting with obstetrics and gynecology doctors. We had them use the checklist-based educational tool for PMS and assessed their menstrual symptoms and work productivity in pre- and post-intervention surveys.

## Methods

This study is a single-arm, pre- and post- intervention study, registered on December 25, 2019, with UMIN Clinical Trials Registry number UMIN000038917. The pre-determined primary outcome is health-related quality of life as measured by the SF-36v2®, which we plan to report elsewhere. In this study, we report changes in their menstrual symptoms and work productivity.

### Participants and the study procedures

We recruited participants via an online social research panel. Inclusion criteria were women aged between 25 and 44 years old who had paid work and were not currently consulting with an obstetrics and gynecology doctor. In Japan, with universal health insurance coverage and the ability to choose any healthcare provider, women usually receive primary care directly from obstetrics and gynecology doctors when they present symptoms related to menstruation or reproductive concerns.

**Fig. 2:**
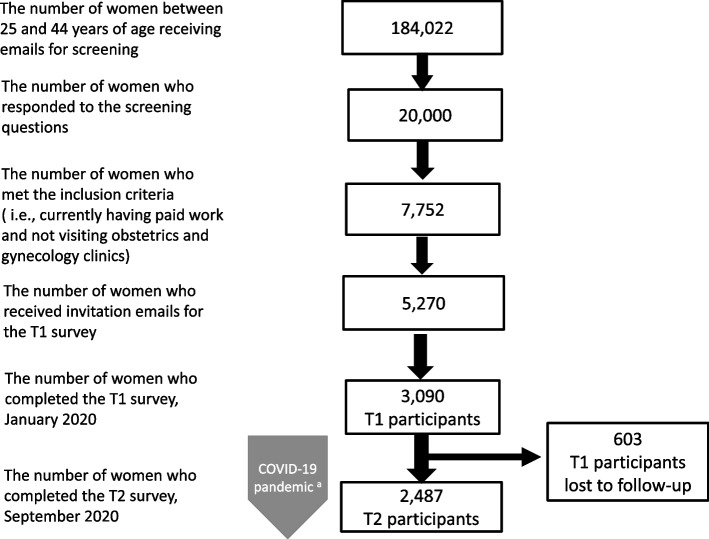
Flow chart of the study procedure. ^a^After the T1 survey (January 6–7, 2020), the first case of COVID-19 was confirmed in Japan on January 15, 2020

An online market research company (Macromill, Tokyo, Japan), which has a nationwide social research panel of more than 1 million registrants, sent prescreening emails for the inclusion criteria to 184,022 randomly selected female registrants aged 25–44 years and then accepted prescreening responses until reaching 20,000 respondents (Fig. 2). Of the 20,000 respondents, there were 7,752 women who reported in the prescreening survey that they were in paid employment and that they did not regularly have access to an obstetrics and gynecology doctor. Of the eligible registrants, 5,270 were randomly selected and received recruitment emails. Finally, 3,090 completed the T1 survey (58.6% participation rate among eligible subjects) during January 6–7, 2020.

All T1 participants were invited to complete the T2 survey, and 2,487 (80.5% follow-up rate) participated during September 7–23, 2020. T2 survey was originally planed in July 2020, but we postponed the T2 survey for two months due to the start of the second wave of the COVID-19 pandemic in Japan. Participants received a coupon (usually worth less than 1 Euro), consistent with Macromill’s procedures for each survey. Participant responses were anonymous.

### T1 survey

Participants provided baseline sociodemographic and lifestyle information; premenstrual status measured by the premenstrual symptoms screening tool (PSST) [14, 15]; premenstrual, menstrual, and intermenstrual symptoms measured by the Menstrual Distress Questionnaire (MDQ) [16, 17]; and responses for the World Health Organisation Health and Work Performance Questionnaire short form (WHO-HPQ) [18–20]. In addition, participants completed an online checklist-based education tool for PMS at the end of the T1 survey (Fig. 1).

#### Sociodemographic and lifestyle information

The online market research company provided participants’ age in years and the prefecture where they lived. Age was categorized as < 35 and ≥ 35 years old. We defined densely populated regions as prefectures with more than 1,500 people per square kilometer of habitable land area [21]: Tokyo, Osaka, Kanagawa, Saitama, Aichi, Kyoto, Hyogo, Fukuoka, Chiba, and Nara Prefecture.

Participants provided information on their marital status (currently married, yes/no), have a child (yes/no), academic background (university education, yes/no), and annual household income: low, < 4 million Japanese Yen (JPY); moderate, 4–6 million JPY; high, ≥ 6 million JPY; and “unknown.” They also reported smoking habits (i.e., current smoker, yes/no), drinking frequency (i.e., habitually drinking three times per week or more, yes/no), and their work hours per week. Work hours were dichotomized at the median (i.e., < 38 or ≥ 38 h per week).

#### Severity of PMS

We used the Japanese version of the PSST [14, 15] to define potential moderate-to-severe PMS or PMDD patients at T1. We defined the participant as a “moderate-to-severe PMS participant (including PMDD)” if she answered “moderate “ or “severe “ for one or more of the items for anger, anxiety, tearful, and depressed mood; for at least four items of the 12 premenstrual symptoms; and at least one of the items regarding interference with activities and relationships, in line with the scoring of the Japanese version [15]. The others were categorized as “none-to-mild PMS participants.”

#### Premenstrual, menstrual, and intermenstrual symptoms

We used the Japanese version of the MDQ [16, 17] to evaluate premenstrual, menstrual, and intermenstrual symptoms. Symptoms were assessed by 46 items on a 4-point scale from 0 (none) to 3 (severe) across eight categories: pain, concentration, behavior change, autonomic reactions, water retention, negative affect, arousal, and control [17]. The scores were summed by premenstrual, menstrual, and intermenstrual periods. The reliability coefficients (i.e., Cronbach alpha) of the premenstrual, menstrual, and intermenstrual scores were high among the present participants: 0.96, 0.97, and 0.97, respectively. A higher score indicated that the participant experienced more severe menstrual-related symptoms.

#### Work productivity

We used the short Japanese version of the WHO-HPQ [18–20] to measure absolute and relative absenteeism (i.e., missed days of work) and presenteeism (i.e., low performance while at work) at their paid work. Participants reported hours for which their employers expected them to work and how many hours they actually worked per month. Absolute absenteeism is the difference between the numbers, ranging from a negative lower bound (i.e., when they worked more than expected) to an upper bound (i.e., the hours they were expected to work). Relative absenteeism is a percentage of expected hours, ranging between a negative number (works more than expected) and 1.0 (always absent).

Absolute presenteeism is participants’ job performance during the past 4 weeks ranging from 0 to 100, where 0 is the lowest job performance and 100 is the highest. Relative presenteeism was obtained by dividing their performance by the usual performance of most workers in the same job. It ranged from 0.25 to 2.0, where 0.25 is the worst relative performance and 2.0 is the best.

#### Online checklist-based education tool for PMS

Participants were instructed to complete the online checklist-based education tool for PMS (Fig. 1) at the end of the T1 survey. It provided basic medical information on PMS (i.e., definition, prevalence) and listed 11 typical PMS symptoms based on the Japanese version of the PSST (Fig. 1a), and it advised the user to consult a doctor if she checked two or more signs on the list (Fig. 1b).

### T2 survey

At T2, participants responded to the MDQ and the WHO-HPQ. They reported whether or not they had visited an obstetrics and gynecology doctor after using the educational tool for PMS (yes, no, or “do not remember”). We categorized those who had visited a doctor as “help-seekers” and those who had not or did not remember as “non-help-seekers.” Of the moderate-to-severe PMS participants, non-help-seekers provided free-text feedback regarding why they had not sought medical help. Only those who had paid work at T2 responded to the WHO-HPQ.

### Text analysis

We analyzed the free-text comments qualitatively to investigate the reasons provided by moderate-to-severe PMS participants for not seeking medical help. Two researchers (CO and EM) separately interpreted, classified, and tallied the items by topic. First, each researcher reviewed respondents’ comments and divided them into individual, single-meaning text fragments. Second, each researcher grouped similar text fragments. Both researchers then discussed the shared meanings of each sorted group and classified them into the broadest but still meaningful categories. Finally, to ensure rigor and consistency of interpretation of the feedback, the researchers discussed disagreements and reached a consensus on all classifications.

### Statistical analyses

To compare the baseline (T1) sociodemographic and lifestyle factors, menstrual symptoms, and work productivity between moderate-to-severe and non-to-mild PMS participants, we used Wilcoxon rank sum tests, chi-squared tests, and Wilcoxon-type tests for trend. For attrition analyses, we examined the difference between T2 participants and those lost to follow-up; we compared the baseline characteristics using Wilcoxon rank sum tests, chi-squared tests, and Wilcoxon-type tests for trend, depending on the type and distribution of the variables.

We performed the following analyses using T2 participants. First, we compared the baseline characteristics between help-seekers and non-help-seekers using chi-square tests to explore factors contributing to help-seeking. Then we conducted univariable and multivariable logistic regression analyses. In multivariable logistic regression analyses, we examined the association of baseline severity of PMS, sociodemographics (i.e., categories for age, having a child, university education, annual household income, and living in a densely-populated area) and lifestyle factors (i.e., categories for smoking habits, drinking frequency, and work hours) with help-seeking using a forced entry method, excluding marital status due to the high multicollinearlity between marital status and having a child. In addition, to assess the MDQ score changes over time by help-seeking and baseline severity of PMS, we performed generalized estimating equation models for panel data among T2 participants, assuming the identity link and Gaussian family. We controlled for all of the aforementioned baseline sociodemographic and lifestyle factors excluding marital status and included interaction terms between the baseline severity of PMS, time, and help-seeking.

As only those who were in paid employment at T2 responded to the WHO-HPQ, we first examined the difference between T2 participants who left paid work and those who remained in paid employment, and then performed generalized estimating equation models for panel data among T2 participants who remained in paid employment, using the same model as for changes in MDQ scores.

A two-sided *P*-value of < 0.05 was used to define statistical significance. All analyses were performed using Stata14-MP (StataCorp LP, College Station, TX, USA).

## Ethics approval

The ethics committee at Akita University Graduate School of Medicine approved the study protocol (no. 2353, approved on December 20, 2019).

## Results

### Baseline characteristics and attrition

Table 1 shows the baseline characteristics of all T1 participants (*n* = 3,090) by the severity of PMS: moderate-to-severe PMS participants (*n* = 497, 16.1%) versus non-to-mild PMS participants (*n* = 2,593, 83.9%). The mean age (interquartile range, IQR) of moderate-to-severe PMS participants was younger, 33 (29–38) years of age, than those with non-to-mild PMS: 35 (30–40) years of age (*P* < 0.001). The proportion of those with a university education was lower among moderate-to-severe PMS participants (44.3%) than non-to-mild PMS participants (50.7%,* P* = 0.009). Moderate-to-severe PMS participants showed much higher MDQ scores throughout menstrual cycles (i.e., premenstrual, menstrual, and intermenstrual periods, all *P* < 0.001). Absolute presenteeism was significantly lower among moderate-to-severe PMS participants than non-to-mild PMS participants, whereas absenteeism and relative presenteeism were similar.

**Table 1:** Baseline (T1) characteristics of all T1 participants by premenstrual syndrome

	None to mild (*n* = 2593)	Moderate to severe (*n* = 497)	*P*
**Demographics**			
Age, median (IQR)	35 (30–40)	33 (29–38)	< 0.001
Married (n, %)	1275 (49.2)	241 (48.5)	0.78
Having a child (n, %)	1065 (41.1)	182 (36.6)	0.06
University education (n, %)	1314 (50.7)	220 (44.3)	0.009
Annual household income (n, %)			
< 4 million JPY	682 (26.3)	152 (30.6)	0.14
≥ 4 & < 6 million JPY	586 (22.6)	109 (21.9)	
≥ 6million JPY	766 (29.5)	141 (28.4)	
Unknown	559 (21.6)	95 (19.1)	
Living in densely populated area (n, %)^a^	1489 (57.4)	274 (55.1)	0.34
**Lifestyles**			
Current smoker (n, %)	305 (11.8)	71 (14.3)	0.12
Habitual drinker (n, %)	485 (18.7)	107 (21.5)	0.14
Working hours per week (median, IQR)	38 (20–40)	35 (20–40)	0.95
≥ 38 h/week (n, %)	1317 (50.8)	244 (49.1)	0.49
**Total scores on the Menstrual Distress Questionnaire, median (IQR)**			
Premenstrual score	20 (9–38)	57 (42–74)	< 0.001
Menstrual score	15 (7–32)	54 (35–74)	< 0.001
Intermenstrual score	4 (1–11)	23 (10–48)	< 0.001
**WHO Health and Work Performance Questionnaire, median (IQR)**			
Absolute absenteeism	0 (− 7 to 18)	0 (− 10 to 22)	0.34
Relative absenteeism	0 (− 0.05 to 0.125)	0 (− 0.08 to 0.225)	0.33
Absolute presenteeism (0–100)	60 (50–70)	50 (40–70)	< 0.001
Relative presenteeism (0.25–2.0)	1 (1–1)	1 (0.83–1)	0.07

*JPY* Japanese Yen, *IQR* interquartile range

^a^Ten prefectures with more than 1500 people per square kilometer of habitable land area

Attrition analyses showed that T2 participants (*n* = 2,487) were significantly older and less likely to be current smokers than T1 participants lost to follow-up (*n* = 603). In contrast, the other sociodemographic factors were similar between groups (Supplementary Table 1). In addition, T2 participants were less likely to have moderate-to-severe PMS (*P* < 0.001), showing lower premenstrual and menstrual MDQ scores than those lost to follow-up (*P* = 0.003 and 0.01, respectively).

### Factors for seeking medical help for PMS

Only 4.9% (121/2487) of the T2 participants sought medical help during the 8-month follow-up period (Table 2). Moderate-to-severe PMS participants were more likely to be help-seekers than non-help-seekers: univariable odds ratio (OR) = 2.36 (95% confidence interval [CI]: 1.56–3.58,* P* < 0.001). In addition, those with high annual household income (i.e., ≥ 6 million JPY) were more likely to have sought medical help (OR = 1.81, 95% CI: 1.08–3.03, *P* = 0.03) compared with those with low household income (i.e., < 4 million JPY). Multivariable logistic regression analyses also showed that moderate-to-severe PMS participants and those having a high annual household income were more likely to have sought medical help after the T1 survey: adjusted OR = 2.27 (95% CI: 1.49–3.46, *P* < 0.001) and 2.07 (95% CI: 1.21–3.53, *P* = 0.01), respectively.

**Table 2:** Baseline (T1) characteristics for medical help-seeking during the follow-up period among T2 participants

	Non-help-seekers (*n* = 2366)	Help-seekers (*n* = 121)	*P*	Univariable logistic regression analyses			Multivariable logistic regression analyses		
				Odds ratio	95% CI	*P*	Odds ratio	95% CI	*P*
**Premenstrual syndrome (n, %)**									
Moderate to severe	**335 (14.2)**	**34 (28.1)**	** < 0.001**	**2.36**	**1.56–3.58**	** < 0.001**	**2.27**	**1.49–3.46**	** < 0.001**
**Demographics (n, %)**									
Age, ≥ 35 years old	1290 (54.5)	55 (45.5)	0.05	0.69	0.48–1.00	0.05	0.72	0.49–1.07	0.10
Married	1153 (48.7)	62 (51.2)	0.59	1.11	0.77**–**1.59	0.59			
Having a child	946 (40.0)	39 (32.2)	0.09	0.71	0.48**–**1.05	0.09	0.75	0.49**–**1.16	0.19
University education	1172 (49.5)	62 (51.2)	0.72	1.07	0.74**–**1.54	0.72	0.98	0.66**–**1.45	0.92
Annual household income									
< 4 million JPY	644 (27.2)	23 (19.0)	0.11	Reference			Reference		
≥ 4 and < 6 million JPY	530 (22.4)	24 (19.8)		1.27	0.71**–**2.27	0.43	1.43	0.79**–**2.58	0.24
≥ 6 million JPY	682 (28.8)	44 (36.4)		**1.81**	**1.08–3.03**	**0.03**	**2.07**	**1.21–3.53**	**0.01**
Unknown	510 (21.6)	30 (24.8)		1.65	0.95**–**2.87	0.08	1.72	0.99**–**3.02	0.06
Living in densely populated area^a^	1362 (57.6)	75 (62.0)	0.34	1.20	0.83**–**1.75	0.34	1.15	0.78**–**1.70	0.48
**Lifestyles (n, %)**									
Current smokers	262 (11.1)	20 (16.5)	0.07	1.59	0.97**–**2.61	0.07	1.62	0.96**–**2.73	0.07
Habitual drinkers	455 (19.2)	29 (24.0)	0.20	1.32	0.86**–**2.03	0.20	1.24	0.79**–**1.94	0.35
Working hours ≥ 38 h/week	1181 (49.9)	64 (52.9)	0.52	1.12	0.78**–**1.62	0.52	1.00	0.68**–**1.47	0.99

Univariable logistic analyses were conducted to calculate the odds to help-seekers compared to non-help-seekers

*JPY* Japanese Yen, *CI* confidence interval

^a^Ten prefectures with more than 1500 people per square kilometer of habitable land area

**Fig. 3:**
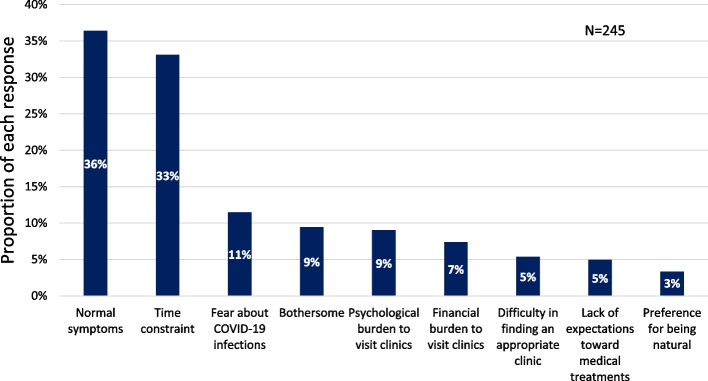
Reasons for not seeking help for premenstrual syndrome (PMS), provided by moderate-to-severe PMS participants

Of the 335 moderate-to-severe PMS participants who had not sought medical help after the T1 survey, 245 provided short text explanations regarding not seeking medical help for their PMS (Fig. 3). Topics identified included “normalization of the symptom,” “time constraint,” “fear about COVID-19 infections,” “bothersome,” “psychological” and “financial burden to visit clinics,” “difficulty in finding an appropriate clinic,” “lack of expectation toward medical treatment,” and “preference for being natural.” Eighty-nine people (36%) characterized their symptoms as being too normal to visit clinics, for example, “This is not a disease,” “My symptoms are not serious,” and “I did not feel medical consultations are necessary for my symptoms.” Eighty-one people (33%) mentioned time constraints, for example, “I am too busy to visit clinics,” “My kids are very young, and I don’t have time,” and “I have missed an opportunity to visit clinics.” Twenty-three people (9%) described medical consultation as just “too much work” or “bothersome.” Avoidance of visiting clinics amid the COVID-19 pandemic was also mentioned in 28 comments (11%). Whereas, 18 comments (7%) indicated financial cost to visit clinics (e.g., “I have no money to spend on medical care”) and 22 comments (9%) described psychological burden, for example, “I will feel embarrassed because doctors do not understand my complaint,” “Visiting clinics makes me feel depressed,” and “Visiting gynecological clinics requires courage.”

### Changes over time in menstrual symptoms and work productivity

**Fig. 4:**
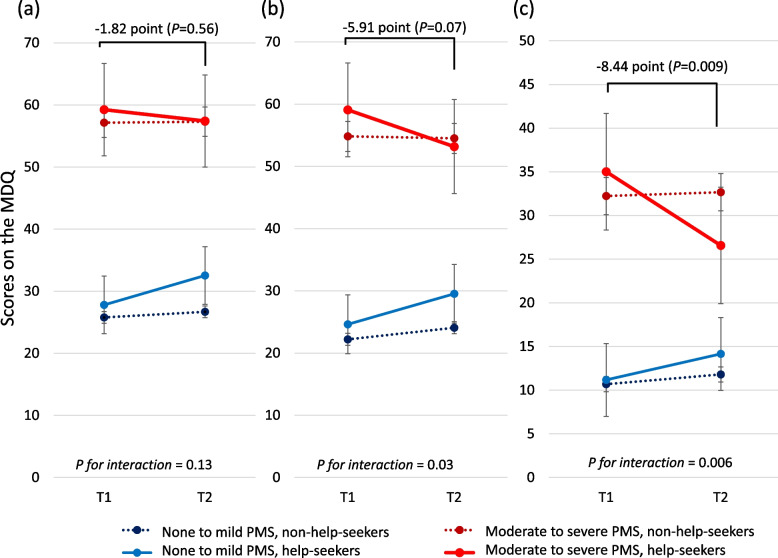
Estimated scores (95% confidence interval) on the Menstrual Distress Questionnaire (MDQ) at T1 and T2. Generalized estimating equation models were used for T2 participants, controlling for the baseline sociodemographic and lifestyle factors and including interaction terms between the baseline severity of PMS, time, and help-seeking. Score changes from T1 to T2 of moderate-to-severe PMS participants who sought medical help are shown. (**a**) Premenstrual MDQ score (**b**) Menstrual MDQ score (**c**) Intermenstrual MDQ score

Generalized estimating equation models for T2 participants (*n* = 2487)　showed that interactions between the baseline PMS severity, help-seeking, and time were not significant for the premenstrual MDQ scores (*P* = 0.13, Fig. 4a), but significant for the menstrual (*P* = 0.03, Fig. 4b) and intermenstrual MDQ score (*P* = 0.006, Fig. 4c). Help-seekers among none-to-mild PMS participants showed significant score increases between T1 and T2 in premenstrual, menstrual, and intermenstrual MDQ scores (Fig. 4a, b, and c). On the other hand, among moderate-to-severe PMS participants, help-seekers showed a − 8.44 (95% CI: − 14.73 to − 2.15) point intermenstrual score change between T1 and T2, whereas non-help-seekers did not show a significant score change (+ 0.43 point, 95% CI: − 1.57 to 2.44) (Fig. 4c). Similarly, help-seekers among moderate-to-severe PMS participants showed a decrease in premenstrual and menstrual scores, − 1.82 (95% CI: − 7.96 to 4.31, *P* = 0.56) and − 5.91 (95% CI: − 12.19 to 0.37, *P* = 0.07), respectively, although it was not significant (Fig. 4a and b). Subscale scores showed similar trends to the total scores; help-seekers among moderate-to-severe PMS participants showed a significant decrease in the intermenstrual behavioral change and negative affect subscale scores (Supplementary Table 2).

**Fig. 5:**
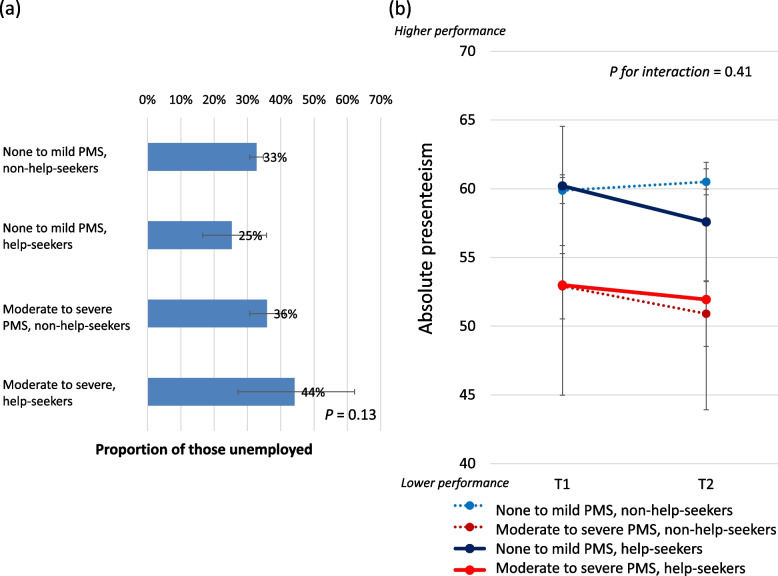
Work productivity of the T2 participants. **a** Proportion of unemployed T2 participants by the severity of PMS and medical help-seeking (**b**) Estimated scores (95% confidence interval) on absolute presenteeism at T1 and T2 Generalized estimating equation models were used for T2 participants who remained in paid employment, controlling for the baseline sociodemographic and lifestyle factors and including interaction terms between the baseline severity of PMS, time, and help-seeking

Of the 2487 participants at T2, 821 (33.0%) did not have paid work. The proportion of those who left paid employment was not significantly different between the baseline PMS severity or help-seeking behavior (*P* = 0.13, Fig. 5a). Participants employed at T2 (*n* = 1666) were significantly older, less likely to be married, have children or smoke, and more likely to have a university education, live in a densely populated area, drink alcohol and work longer hours at baseline than their unemployed counterparts, although baseline MDQ and WHO-HPQ scores did not differ significantly between groups (Supplementary Table 3). Based on the difference in absolute presenteeism by the severity of PMS (Table 1), we examined interactions between baseline PMS severity, help-seeking, and time for the change in absolute presenteeism, but found no significant interactions (*P* = 0.41, Fig. 5b).

## Discussion

We had reproductive-aged women use a checklist-based educational tool for PMS and assessed their menstrual symptoms and work productivity in a single-arm, pre- and post-intervention study. Our short-term, longitudinal study suggests that seeking medical help following PMS awareness using the checklist-based educational tool reduces some symptoms, at least a significant improvement in intermenstrual symptoms in women with moderate-to-severe PMS. Moderate-to-severe PMS participants felt more menstrual distress throughout their cycles and reported lower work productivity than non-to-mild PMS participants. Given that nearly one-in-six working women of reproductive age experience considerable symptoms related to menstruation, building PMS awareness and encouraging medical help-seeking could be a first step to improving their symptoms.

Of the 369 women who completed the checklist-based educational tool and received a screening diagnosis of moderate-to-severe PMS, only 34 (9%) consulted a doctor (Table 2). Although this one-armed study could not measure the effects of the tool on medical help-seeking, 9% during the 8-month follow-up might not be low although the reason for the visit might include other obstetric and gynecological reasons. Given previous studies showing natural courses, only 5% of moderate-to-severe PMS patients are known to receive medical treatment [7]. In addition, 7% of the young working population in Korea was diagnosed at medical facilities as having PMS during an 8-year study period [22], which could approximate to an annual consultation rate of about 3%, assuming a PMS prevalence of 32% among Korean women [23]. Furthermore, in consideration of the follow-up period amid the COVID-19 pandemic, the rate could have been even higher; 11% of the non-help-seekers listed a fear of COVID-19 infection as a reason for not visiting a doctor (Fig. 3). As a future direction, randomized two-group comparisons are needed to confirm the effects of PMS awareness on medical help-seeking.

Among moderate-to-severe PMS participants, those who sought medical help showed significantly lower intermenstrual MDQ scores at T2 (Fig. 4c). Since moderate-to-severe PMS participants felt uncomfortable throughout their menstrual cycles, a significant improvement of intermenstrual symptoms would benefit them. Although the observed improvement may be partly due to the correlation between seasonal and premenstrual symptoms [24], the lack of improvement in non-help-seekers would support our finding that help-seeking behaviour would have some effect on improvement in MDQ scores.

Similarly, premenstrual and menstrual MDQ scores showed a trend towards improvement, but were not significant (Fig. 4a & b). The insignificant results may be due to the small number of help-seekers and the resultant lack of statistical power. On the other hand, the insignificant results highlight the future necessity to evaluate the effects of treatments on a specific treatment basis. For example, patients suffering from PMS or PMDD would visit genecologists but less than 3% of obstetricians and gynecologists in Japan consider selective serotonin reuptake inhibitors (SSRIs) as a first-line option [25], although SSRIs are standard pharmacotherapy in other countries [1, 5, 6]. In addition, female doctors were less likely to prescribe SSRIs, possibly because of negative patient attitudes towards antidepressants in Japan [26]. Therefore, there is a discrepancy between clinical guidelines and actual clinical practice. Future studies should focus on the treatments patients actually receive and measure real-world effectiveness.

Most women did not seek medical help. Low-income women were less likely to have consulted a doctor (Table 2), which is consistent with previous behavioral models presenting a relationship between financial factors and health service use [27, 28]. Our text analyses additionally revealed that other enabling factors (e.g., time) and needs [28] (e.g., subjective symptoms) played an essential role in help-seeking behaviors for PMS (Fig. 3). Although the checklist-based educational tool was designed to help people understand their need for treatment, a substantial proportion of moderate-to-severe PMS participants felt less need, and their symptoms did not change over time (Fig. 4). Since comprehension of PMS could alleviate some symptoms through positive reframing [29], providing more self-care information, such as exercise, nutrition, cognitive behavioral therapy, or self-medication products, and increasing self-efficacy might benefit those not seeking medical help. Recent studies of self-care apps [30–32] for PMS and menstrual-related symptoms are promising methods for future PMS management. Another approach to benefit those not seeking medical help was to lower the barrier to visit clinics (i.e., time, cost, and psychological factor). For example, annual mandatory health checkups in Japan at workplaces [33] would be a good opportunity to screen for PMS and providing female employees with medical advice.

PMS symptoms affect working productivity and sometimes lead to absences from work [34]. We did not find absenteeism among moderate-to-severe PMS participants, but found degradation in absolute presenteeism (Table 1), suggesting that they were not satisfied with their job performance as much as those with non-to-mild PMS. Since we measured presenteeism over the previous 4 weeks, presenteeism at the premenstrual phase might have been even worse; a qualitative study reported that female workers with PMDD felt guilty and overcompensated in their work when the symptoms disappeared [35]. Given the high prevalence of PMS and the greater work participation of women in society, the impact of PMS on the labor market should not be underestimated.

However, 33% of the T2 participants left paid employment (Fig. 5a). As their presenteeism was not measured at the follow-up survey in September 2020, we could not assess changes in absolute presenteeism over time with a sufficient sample size. This surprisingly high unemployment rate agrees with a nationwide survey investigating the effects of the COVID-19 pandemic on employment and life in Japan [36]; 26% of women experienced a massive change in their work (e.g., disemployment, turnover, loss of time) from April to November 2020, compared with 19% of men. Irregular employment and having a child are known to be risk factors for leaving paid work in the midst of the COVID-19 pandemic [36], which is also consistent with our findings (Supplementary Table 3). In our study, the percentage of women who left work was not significantly different based on the severity of PMS (Fig. 5a), but previous studies observed an association between presenteeism and future sickness absence [37]. Long-term evaluation of PMS on employment is needed.

There are several limitations to this study. First, this is a single-arm, pre-post study, and thus the temporal relationships we observed could be caused by other changes irrelevant to the intervention. To confirm our findings, randomized controlled trials would be needed. Second, attrition decreased the statistical power and limited the generalizability of our findings. Although the follow-up rate was high (i.e., > 80%), T1 participants lost to follow-up were significantly younger and more likely to be a smoker and have premenstrual symptoms (Supplementary Table 1). Thus, the percentage of help-seeking participants among the total sample is unknown. However, our results would not be heavily biased since we appropriately adjusted for the study participants’ backgrounds to estimate the factors for help-seeking and changes in menstrual symptoms. Third, a small number of participants seeking medical help during the COVID-19 pandemic might have induced beta errors for the premenstrual and menstrual score changes over time. Fourth, we could not obtain user histories of the checklist-based educational tool of each participant. Thus we assumed that all the participants correctly responded to the checklist and that all the “moderate-to-severe PMS participants” had received advice to consult doctors through the tool. Fifth, we included participants who did not see an obstetrician and gynaecologist at T1, regardless of their clinical history. Thus, there is a possibility that women without menstruation (e.g., early menopause) may have been included as participants, although we assumed that they were excluded by the inclusion criteria that participants did not have regular access to an obstetrician and gynaecologist. Also, those who had previously visited clinics would be more likely to consult a doctor than those who had not, and the proportion of those who consulted a doctor after intervention might be overestimated. Finally, this study was conducted in Japan using a social research panel, which could have caused selection bias. Since participants in the internet panel survey are generally highly educated [38], educational effects observed in the present study might be overestimated. Future studies should include other groups or cultures.

## Conclusion

Medical help-seeking after PMS awareness alleviated intermenstrual symptoms in women with moderate-to-severe PMS. However, most of the women with moderate-to-severe PMS in our study did not seek medical help despite the educational intervention. Since women with moderate-to-severe PMS presented more severe symptoms throughout a menstrual cycle and lower working productivity, further research is necessary to benefit the majority of women feeling less need for medical help.

### Supplementary Information

**Supplementary Material 1.**

## Data Availability

The datasets generated and analyzed during the current study are not publicly available due to participants’ privacy, but are available from the corresponding author upon reasonable request.

## Appendix

### Common symptoms of premenstrual syndrome

**Table:**

Emotional symptoms	Physical symptoms
• depression	• thirst and appetite changes (food cravings)
• angry outbursts	• breast tendrness
• irritability	• bloating and weight gain
• crying spells	• headache
• anxiety	• swelling of the hands or feet
• confusion	• aches and pains
• social withdrawal	• fatigue
• poor concentration	• skin problems
• insomnia	• gastrointestinal symptoms
• increased nap taking	• abdominal pain
changes in sexual desire	

Reference: The American College of Obstetricians and Gynecologists [2]

## Abbreviations

PMS: Premenstrual syndrome
MDQ: Menstrual Distress Questionnaire
PSST: Premenstrual symptoms screening tool
SSRI: Selective serotonin reuptake inhibitor
WHO-HPQ: World Health Organisation Health and Work Performance Questionnaire
JPY: Japanese Yen
OR: Odds ratio
CI: Confidence interval
PMDD: Premenstrual dysphoric disorder
