# Supplementary material for: Changes in menstrual symptoms and work productivity after checklist-based education for premenstrual syndrome: an 8-month follow-up of a single-arm study in Japan
Source: BMC Womens Health. 2024 Apr 15;24:242. doi: 10.1186/s12905-024-03067-2 (PMC11017586; doi:10.1186/s12905-024-03067-2)
Supplement: Supplementary file 1 — Supplementary Material 1. [file 12905_2024_3067_MOESM1_ESM.docx]

|  | All  T1 participants (n = 3090) |  | T1 participants lost to follow-up (n = 603) | T2 participants (n = 2487) | *P* |
| --- | --- | --- | --- | --- | --- |
| **Demographics** |  |  |  |  |  |
| Age, median (IQR) | 35 (30–40) |  | 33 (29–38) | 35 (31–40) | <0.001 |
| Married (n, %) | 1516 (49.1) |  | 301(49.9) | 1215 (48.9) | 0.64 |
| Having a child (n, %) | 1247 (40.4) |  | 262 (43.4) | 985 (39.6) | 0.08 |
| University education (n, %) | 1534 (49.6) |  | 300 (49.8) | 1234 (49.6) | 0.95 |
| Annual household income (n, %) |  |  |  |  | 0.97 |
| <4 million JPY | 834 (27.0) |  | 167 (27.7) | 667 (26.8) |  |
| ≥4 & <6 million JPY | 695 (22.5) |  | 141 (23.4) | 554 (22.3) |  |
| ≥6million JPY | 907 (29.4) |  | 181 (30.0) | 726 (29.2) |  |
| Unknown | 654 (21.2) |  | 114 (18.9) | 540 (21.7) |  |
| Living in densely populated area (n, %)^a^ | 1763 (57.1) |  | 326 (54.1) | 1437 (57.8) | 0.10 |
| **Lifestyles** |  |  |  |  |  |
| Current smokers (n, %) | 376 (12.2) |  | 94 (15.6) | 282(11.3) | 0.004 |
| Habitual drinkers (n, %) | 592 (19.2) |  | 108 (17.9) | 484(19.5) | 0.38 |
| Working hours per week (median, IQR) | 38 (20–40） |  | 40 (21–40) | 38 (20–40) | 0.10 |
| ≥38 hours/week (n, %) | 1561 (50.5) |  | 316 (52.4) | 1245 (50.1) | 0.30 |
| **Premenstrual syndrome** |  |  |  |  |  |
| None to mild (n, %) | 2593 (83.9) |  | 475 (78.8) | 2118 (85.2) | <0.001 |
| Moderate to severe (n, %) | 497 (16.1) |  | 128 (21.2) | 369 (14.8) |  |
| **Total scores on the Menstrual Distress Questionnaire, median (IQR)** | | | |  |  |
| Premenstrual score | 25 (11–47) |  | 28 (13–51) | 24 (11–46) | 0.003 |
| Menstrual score | 19 (8–41) |  | 22 (10–46) | 19 (8–40) | 0.01 |
| Intermenstrual score | 5 (1–17) |  | 5 (1–17) | 5 (1–17) | 0.56 |
| **WHO Health and Work Performance Questionnaire, median (IQR)** | | | |  |  |
| Absolute absenteeism | 0 (−8 to 20) |  | 0 (−8 to 20) | 0 (−8 to 20) | 0.59 |
| Relative absenteeism | 0 (−0.06 to 0.125) |  | 0 (−0.05 to 0.17) | 0 (−0.06 to 0.125) | 0.56 |
| Absolute presenteeism (0–100) | 60 (50–70) |  | 60 (50–70) | 60 (50–70) | 0.88 |
| Relative presenteeism (0.25–2.0) | 1 (0.9–1) |  | 1(1–1) | 1 (0.9–1) | 0.16 |

Supplementary Table 1. Baseline (T1) characteristics of all T1 participants, those lost to follow-up, and T2 participants

IQR, interquartile range; JPY, Japanese Yen; PMS, Premenstrual syndrome

Statistical tests compare the T2 participants with those lost to follow-up.

Supplementary Table 2. The Menstrual Distress Questionnaire score differences among moderate-to-severe PMS participants by help-seeking and time

|  | Premenstrual | | | | |  | Menstrual | | | | |  | Intermenstrual | | | | |
| --- | --- | --- | --- | --- | --- | --- | --- | --- | --- | --- | --- | --- | --- | --- | --- | --- | --- |
|  | Coefficient | *P* | 95%CI | | |  | Coefficient | *P* | 95%CI | | |  | Coefficient | *P* | 95%CI | | |
| **Total** |  |  |  |  |  |  |  |  |  |  |  |  |  |  |  |  |  |
| Help-seeking at T2 | 0.12 | 0.98 | -7.67 | to | 7.90 |  | -1.34 | 0.74 | -9.27 | to | 6.58 |  | -6.10 | 0.09 | -13.10 | to | 0.90 |
| T1-T2 change among help-seekers | -1.82 | 0.56 | -7.96 | to | 4.31 |  | -5.91 | 0.07 | -12.19 | to | 0.37 |  | **-8.44** | **0.01** | **-14.73** | to | **-2.15** |
| *Ps for the interaction term* | 0.13 |  |  |  |  |  | 0.03 |  |  |  |  |  | **0.01** |  |  |  |  |
| **Pain** |  |  |  |  |  |  |  |  |  |  |  |  |  |  |  |  |  |
| Help-seeking at T2 | 0.00 | 1.00 | -1.46 | to | 1.45 |  | 0.22 | 0.78 | -1.27 | to | 1.70 |  | -0.62 | 0.31 | -1.83 | to | 0.58 |
| T1-T2 change among help-seekers | -0.97 | 0.09 | -2.08 | to | 0.14 |  | -0.79 | 0.17 | -1.91 | to | 0.33 |  | -1.35 | 0.02 | -2.46 | to | -0.25 |
| *Ps for the interaction term* | 0.03 |  |  |  |  |  | 0.07 |  |  |  |  |  | 0.06 |  |  |  |  |
| **Concentration** |  |  |  |  |  |  |  |  |  |  |  |  |  |  |  |  |  |
| Help-seeking at T2 | 0.06 | 0.94 | -1.58 | to | 1.71 |  | -0.85 | 0.31 | -2.50 | to | 0.79 |  | -1.56 | 0.02 | -2.91 | to | -0.21 |
| T1-T2 change among help-seekers | -0.03 | 0.97 | -1.43 | to | 1.38 |  | -0.97 | 0.16 | -2.33 | to | 0.39 |  | -1.15 | 0.07 | -2.40 | to | 0.10 |
| *Ps for the interaction term* | 0.31 |  |  |  |  |  | 0.02 |  |  |  |  |  | 0.07 |  |  |  |  |
| **Behavioral change** |  |  |  |  |  |  |  |  |  |  |  |  |  |  |  |  |  |
| Help-seeking at T2 | -0.37 | 0.56 | -1.63 | to | 0.88 |  | -0.08 | 0.91 | -1.39 | to | 1.24 |  | -0.81 | 0.10 | -1.78 | to | 0.16 |
| T1-T2 change among help-seekers | -0.53 | 0.33 | -1.58 | to | 0.52 |  | -0.85 | 0.11 | -1.91 | to | 0.21 |  | **-1.03** | **0.03** | **-1.94** | to | **-0.12** |
| *Ps for the interaction term* | 0.05 |  |  |  |  |  | 0.05 |  |  |  |  |  | **0.02** |  |  |  |  |
| **Autonomic reactions** |  |  |  |  |  |  |  |  |  |  |  |  |  |  |  |  |  |
| Help-seeking at T2 | 0.07 | 0.85 | -0.67 | to | 0.81 |  | 0.61 | 0.13 | -0.17 | to | 1.39 |  | -0.44 | 0.17 | -1.05 | to | 0.18 |
| T1-T2 change among help-seekers | 0.09 | 0.80 | -0.58 | to | 0.76 |  | -0.12 | 0.74 | -0.82 | to | 0.59 |  | -0.41 | 0.18 | -1.02 | to | 0.19 |
| *Ps for the interaction term* | 0.50 |  |  |  |  |  | 0.45 |  |  |  |  |  | **0.04** |  |  |  |  |
| **Water retention** |  |  |  |  |  |  |  |  |  |  |  |  |  |  |  |  |  |
| Help-seeking at T2 | 0.19 | 0.70 | -0.77 | to | 1.15 |  | -0.28 | 0.57 | -1.24 | to | 0.68 |  | -0.51 | 0.16 | -1.23 | to | 0.20 |
| T1-T2 change among help-seekers | 0.03 | 0.94 | -0.74 | to | 0.80 |  | -0.65 | 0.11 | -1.43 | to | 0.14 |  | -0.62 | 0.07 | -1.30 | to | 0.06 |
| *Ps for the interaction term* | 0.14 |  |  |  |  |  | 0.23 |  |  |  |  |  | 0.20 |  |  |  |  |
| **Negative affect** |  |  |  |  |  |  |  |  |  |  |  |  |  |  |  |  |  |
| Help-seeking at T2 | -0.66 | 0.49 | -2.53 | to | 1.21 |  | -0.22 | 0.82 | -2.03 | to | 1.60 |  | -1.45 | 0.05 | -2.91 | to | 0.01 |
| T1-T2 change among help-seekers | -1.12 | 0.14 | -2.59 | to | 0.35 |  | -1.32 | 0.08 | -2.81 | to | 0.16 |  | **-2.56** | **0** | **-3.92** | to | **-1.20** |
| *Ps for the interaction term* | 0.05 |  |  |  |  |  | 0.21 |  |  |  |  |  | **0** |  |  |  |  |
| **Arousal** |  |  |  |  |  |  |  |  |  |  |  |  |  |  |  |  |  |
| Help-seeking at T2 | -0.18 | 0.69 | -1.06 | to | 0.71 |  | -0.67 | 0.09 | -1.46 | to | 0.11 |  | 0.12 | 0.81 | -0.86 | to | 1.09 |
| T1-T2 change among help-seekers | -0.21 | 0.67 | -1.14 | to | 0.73 |  | -0.47 | 0.26 | -1.29 | to | 0.35 |  | -0.50 | 0.28 | -1.41 | to | 0.41 |
| *Ps for the interaction term* | 0.63 |  |  |  |  |  | 0.09 |  |  |  |  |  | **0.035** |  |  |  |  |
| **Control** |  |  |  |  |  |  |  |  |  |  |  |  |  |  |  |  |  |
| Help-seeking at T2 | 1.01 | 0.05 | 0.02 | to | 2.00 |  | -0.07 | 0.90 | -1.07 | to | 0.94 |  | -0.82 | 0.07 | -1.72 | to | 0.08 |
| T1-T2 change among help-seekers | 0.91 | 0.05 | 0.00 | to | 1.82 |  | -0.74 | 0.11 | -1.64 | to | 0.17 |  | -0.82 | 0.06 | -1.67 | to | 0.02 |
| *Ps for the interaction term* | 0.37 |  |  |  |  |  | 0.04 |  |  |  |  |  | **0.05** |  |  |  |  |

Generalized estimating equation models were used, controlling for the baseline sociodemographic and lifestyle factors and including interaction terms between the baseline severity of PMS, time, and help-seeking. Score differences between help-seekers and non-help-seekers (reference) at T2 and score changes from T1 to T2 of moderate-to-severe PMS participants who sought medical help are shown.

|  | All  T2 participants (n = 2487) |  | Unemployed  T2 participants (n = 821) | Employed  T2 participants (n = 1666) | *P* |
| --- | --- | --- | --- | --- | --- |
| **Demographics** |  |  |  |  |  |
| Age, median (IQR) | 35 (31-40) |  | 35 (30-40) | 36 (31-40) | 0.001 |
| Married (n, %) | 1215(48.9) |  | 456 (55.5) | 759 (45.6) | <0.001 |
| Having a child (n, %) | 985(39.6) |  | 361 (44.0) | 624 (37.5) | <0.001 |
| University education (n, %) | 1234(49.6) |  | 375 (45.7) | 859 (51.6) | 0.006 |
| Annual household income |  |  |  |  | 0.24 |
| <4 million JPY (n, %) | 667(26.8) |  | 226 (27.5) | 441 (26.5) |  |
| ≥4 & <6 million JPY | 554(22.3) |  | 194 (23.6) | 360 (21.6) |  |
| ≥6million JPY | 726(29.2) |  | 225 (27.4) | 501 (30.1) |  |
| Unknown (n, %) | 540(21.7) |  | 176 (21.4) | 364 (21.8) |  |
| Living in densely populated area | 1437 (57.8) |  | 447 (54.4) | 990 (59.4) | 0.02 |
| **Lifestyles** |  |  |  |  |  |
| Current smokers (n, %) | 282(11.3) |  | 88 (10.7) | 194 (11.6) | 0.49 |
| Habitual drinkers (n, %) | 484(19.5) |  | 138 (16.8) | 346 (20.8) | 0.02 |
| Working hours per week (median, IQR) | 38 (20-40) |  | 35 (20-40) | 38 (21-40) | 0.003 |
| >=38 hours/week (n, %) | 1,245 (50.1) |  | 392 (47.7) | 853 (51.2) | 0.10 |
| **Premenstrual syndrome** |  |  |  |  |  |
| None to mild (n, %) | 2118 (85.2) |  | 686 (83.6) | 1432 (86.0) | 0.11 |
| Moderate to severe (n, %) | 369 (14.8) |  | 135 (16.4) | 234 (14.0) |  |
| **Total scores on the Menstrual Distress Questionnaire, median (IQR)** | | | |  |  |
| Premenstrual score | 24 (11-46) |  | 27 (11-48) | 24 (10-46) | 0.05 |
| Menstrual score | 19 (8-40) |  | 18 (8-43) | 19 (8-39) | 0.33 |
| Intermenstrual score | 5 (1-17) |  | 5 (1-21) | 5 (1-15) | 0.07 |
| **WHO Health and Work Performance Questionnaire, median (IQR)** | | | |  |  |
| Absolute absenteeism | 0 (-8-20) |  | 0 (-8-20) | 0 (-8-20) | 0.88 |
| Relative absenteeism | 0 (-0.06-0.125) |  | 0 (-0.06-0.125) | 0 (-0.06-0.125) | 0.82 |
| Absolute presenteeism (0-100) | 60 (50-70) |  | 60 (50-70) | 60 (50-70) | 0.23 |
| Relative presenteeism (0.25-2.0) | 1 (0.9-1) |  | 1 (1-1) | 1 (0.9-1) | 0.34 |

Supplementary Table 3. Baseline (T1) characteristics of all T2 participants, those unemployed at T2, and those employed at T2

IQR, interquartile range; JPY, Japanese Yen

Statistical tests compare the employed T2 participants with the unemployed T2 participants.
